# Beliefs about unmet interpersonal needs mediate the relation between conflictual family relations and borderline personality features in young adult females

**DOI:** 10.1186/2051-6673-1-11

**Published:** 2014-08-18

**Authors:** Allison Kalpakci, Amanda Venta, Carla Sharp

**Affiliations:** Department of Psychology, University of Houston, 126 Heyne, Houston, TX 77024 USA

**Keywords:** Family conflict, Borderline personality disorder, Interpersonal Psychological Theory, Multiple mediation, Thwarted belongingness, Perceived burdensomeness

## Abstract

**Background:**

Central to most theories of borderline personality disorder (BPD) is the notion that the family environment interacts with genetically-based vulnerabilities to influence the development of BPD, with particular attention given to risk conferred by conflictual familial relations. However, the extent to which family conflict may relate to the development of BPD via related interpersonal beliefs is currently unknown. This study sought to test the hypothesis that the concurrent relation between conflictual family relations and borderline features in female college students is explained by beliefs associated with real or perceived unmet interpersonal needs (captured by Joiner’s [2005] Interpersonal Psychological Theory, specifically thwarted belongingness and perceived burdensomeness).

**Method:**

The sample included 267 female undergraduates ages 18–25 years (*M* = 20.86; *SD* = 1.80). Level of borderline personality features, unmet interpersonal needs, and family conflict were assessed.

**Results:**

Bivariate analyses revealed significant relations between both thwarted belongingness and perceived burdensomeness, conflictual family relations, and borderline features. Multivariate analyses revealed that thwarted belongingness and perceived burdensomeness both mediated the relation between family conflict and borderline personality features, thus supporting a multiple mediation model.

**Conclusions:**

This cross-sectional study is a preliminary step towards confirming the broad theoretical hypothesis that conflictual family relations relate to beliefs about thwarted belongingness and perceived burdensomeness, which, in turn, relate to borderline personality pathology. Limitations and areas of future research are discussed.

## Background

Borderline personality disorder (BPD) is a serious psychiatric condition associated with marked dysfunction across affective, interpersonal, and cognitive domains [1]. Individuals with the disorder are disproportionately represented in inpatient psychiatric hospitalizations [2, 3] and it has been estimated that nearly 10% of those with BPD complete suicide [4, 5].

Given associations between this disorder and some poor outcomes, including suicide and hospitalization [2–5], theorists have prioritized study of its etiological origins, culminating in the consensus that BPD likely arises from complex transactions between biological and socio-environmental factors. One socio-environmental factor that has been identified in both DBT-oriented [6–8] and mentalization-based [9–11] developmental theories of BPD is the role of the family environment. For instance, Linehan [8] emphasizes a family system that neglects, mislabels, criticizes, negates, or dismisses emotional experiences and/or expressions, and Fonagy and colleagues [9, 10] emphasize insecure and disorganized attachment relations as central developmental processes.

From these theoretical origins, several studies have aimed to empirically identify family characteristics that may relate to the development of BPD. Results from this extensive research base demonstrate that individuals with BPD report high rates of family adversity including past sexual and/or physical abuse [12–17] neglect [15, 17–19] denial of thoughts and feelings [18–20] and inconsistent treatment by caregivers [18, 21].

Additionally, conflictual family relations have been identified as a well-documented key correlate of BPD. Indeed, several retrospective and prospective studies have demonstrated associations between family conflict and borderline features [22–31]. Although this previous research points to an important link between conflictual family relations and BPD, these studies solely measured family conflict that occurred in participants’ childhood (assessed either prospectively or retrospectively), failing to account for how *current* family conflict may relate to borderline pathology in young adulthood. Though childhood represents a developmental stage when family environment is particularly impactful, family dynamics during young adulthood continue to influence psychosocial functioning [32–35]. Thus, our understanding of how family conflict continues to relate to borderline pathology in college-aged adults is limited, demonstrating the need for further research.

Moreover, no studies have identified how conflictual family relations relate mechanistically to borderline features in this population. The two aforementioned theories of the development of BPD speak to this question. In Linehan’s model, the aggregation of chronic invalidating responses towards a child’s emotions leads to a pattern of self-invalidation, promoting intense feelings of abandonment and subsequent dysregulated emotional responses [8]. In Fonagy and colleagues’ [9, 10] account, insecure attachment leads to maladaptive mentalizing in which the reflection on self-other relatedness becomes distorted, thereby disrupting the development of a coherent self. Together, these theories suggest that conflictual family relations may relate to BPD, in part, through maladaptive affective, cognitive, and interpersonal processes related to themes of being abandoned, unwanted, and unlovable—pointing to an empirically testable mediational model. However, no studies have investigated whether beliefs regarding unmet interpersonal needs might explain the relation between conflictual family relations and borderline features, partly due to the lack of measures designed to assess these beliefs.

Joiner’s [36] Interpersonal Psychological Theory offers a helpful theoretical and measurement framework in this regard, by operationalizing beliefs about unmet interpersonal needs [37] in two broad domains: thwarted belongingness and perceived burdensomeness. In this theory, it is posited that an unmet need to belong leads to thwarted belongingness [36, 37], internalized perceptions of alienation and beliefs of not being cared about. The Interpersonal Needs Questionnaire INQ; [38], operationalizes this construct using statements like “These days, I feel disconnected from other people” and “These days, I feel like I belong” (reverse coded). The second domain in Joiner’s theory, perceived burdensomeness, refers to the individual’s belief that those around him or her would be better off without him/her [36, 37]. In other words, the individual believes that he or she does not contribute to others and rather interferes with their success, happiness, etc. The INQ operationalizes this construct using statements like “These days the people in my life would be happier without me”, and “These days I think I matter to the people in my life” (reverse coded). As the only existing measure specifically assessing beliefs associated with these unmet interpersonal needs, the INQ may serve as a valuable addition to research seeking to empirically test whether conflictual family relations relate to BPD through maladaptive beliefs, as is supported by the aforementioned theoretical models of the development of BPD. Moreover, the applicability of the Interpersonal Psychological Theory to borderline personality pathology has already been established in previous research demonstrating associations between beliefs about thwarted belongingness and burdensomeness and borderline personality symptomatology [37, 39, 40]. This provides further reason to examine the potential mechanistic role of these maladaptive beliefs in the relation between family conflict and borderline features.

Against this background, it was the aim of the current study to address the gap in the literature regarding the relation between current family conflict and borderline personality features in college-aged individuals and to examine potential mechanisms in this relation. To this end, we tested the hypothesis that family conflict relates to borderline features cross-sectionally and that beliefs about thwarted belongingness and burdensomeness mediate the relation between conflictual family relations and borderline personality features in female undergraduates Identifying the mechanisms by which conflictual family relations relate to borderline features is of particular therapeutic value, as thwarted belongingness and perceived burdensomeness are beliefs suggested to be “dynamic and amenable to therapeutic change” [37].

## Methods

### Participants

Data were collected from 267 female undergraduate students at The University of Houston, a large and diverse university in the Southwestern United States. Participants were recruited via a mass email advertising this online study to undergraduate students enrolled in at least one Psychology course. The recruitment email was sent from the Department of Psychology and participants self-selected to participate in this study by following a hyperlink to the University’s online survey system. Inclusion criteria were English fluency and age between 18 and 25. Participants were informed of the inclusion criteria in a cover letter and were instructed to self-exclude if the aforementioned criteria were not met. The number of males who participated in the study was very low, so they were therefore excluded from the study. The mean age in this sample was 20.86 (*SD* = 1.80). The self-identified ethnic breakdown was as follows: Black = 18.3% (*n* = 63), White = 22.0% (*n* = 76), Hispanic = 24.1% (*n* = 83), Asian = 26.1% (*n =* 90), Middle Eastern = 4.1% (*n* = 14), and Other = 5.5% (*n* = 19). This study was approved by the University of Houston Institutional Review Board and informed consent was provided. Participants completed questionnaires via a web-based program and were compensated with research credit.

### Measures

#### Borderline personality features

The Personality Assessment Inventory (Borderline Scale, PAI-BOR; Morey, 2007) is a dimensional measure of borderline personality symptomology, with 24 items that are rated on a four-point scale. Items assess four empirically derived subfactors of BPD including affective instability, identity problems, negative relationships, and self-harm. Adequate psychometric properties have been reported for the measure [41]. Internal consistency, as measured by Cronbach’s alpha, was α = 0.88 in this study.

#### Thwarted belongingness and perceived burdensomeness

Beliefs associated with thwarted belongingness and perceived burdensomeness were assessed with the Interpersonal Needs Questionnaire INQ; [38], a self-report questionnaire. The INQ consists of 25 items measured on a seven-point Likert scale, with higher numbers indicating greater endorsement. Ten of the items pertain to belongingness (e.g., “These days, I feel like I belong”), while the other fifteen items pertain to perceived burdensomeness (e.g., “These days the people in my life would be better off if I were gone”). In this study, internal consistency, as measured by Cronbach’s alpha, was α = 0.94.

#### Family conflict

The Conflict Behavior Questionnaire CBQ-20 [42] is a 20-item self-report measure intended to capture conflictual aspects of parent–child relationships. Items are rated as *true* or *false* and include statements like “My parents don’t understand me”, “My parents put me down”, “When I state my own opinion, my parents get upset”, and “The talks we have are frustrating”. Higher scores suggest poorer relationship perception and higher degrees of conflict. Construct validity is supported by findings that distressed families reported significantly higher scores on this scale than non-distressed families [43]. Internal consistency, as measured by Cronbach’s alpha, was α = 0.94 in this study.

## Results

### Preliminary analyses

In this sample, the mean total score for borderline features (PAI-BOR) was 51.30 (*SD* = 11.40) and the mean total score for family conflict (CBQ) was 4.51 (*SD* = 5.60). The mean levels of thwarted belongingness and perceived burdensomeness (INQ) were 25.10 (*SD* = 12.13) and 34.51 (*SD* = 15.14), respectively. Age at time of assessment was not significantly related to any of the study measures. At the bivariate level, self-reported borderline features (PAI-BOR) were significantly correlated with family conflict (CBQ; *r* = .47, *p* = 0.000), thwarted belongingness (INQ; *r* = .59, *p* = 0.000), and perceived burdensomeness (INQ; *r* = .59, *p* = 0.000), such that greater borderline symptomology was associated with greater thwarted belongingness and perceived burdensomeness and a more conflictual family relations.

### Mediational analyses

**Figure 1:**
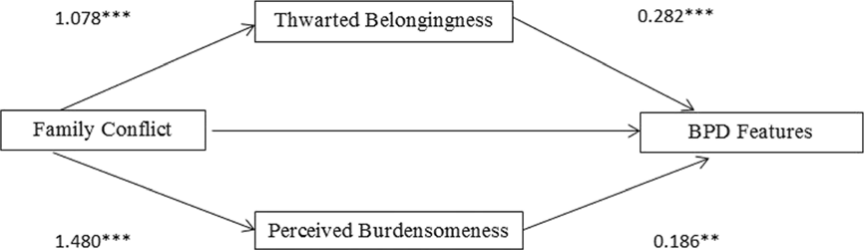
**Multiple mediation model exploring the effect of invalidating family environment on borderline personality features through proposed mediators of thwarted belongingness and perceived burdensomeness.** *Note*. Values are unstandardized path coefficients. Family Conflict = Total scale from the Conflict Behavior Questionnaire; Thwarted Belongingness = Thwarted belongingness scale from the Interpersonal Needs Questionnaire; Perceived Burdensomeness = Perceived burdensomeness scale from the Interpersonal Needs Questionnaire; BPD Features = Total score of the Borderline scale from the Personality Assessment Inventory. * *p* < .05. **p < .01. ***p < .001.

The aim of this study was to determine whether beliefs about perceived burdensomeness and/or thwarted belongingness explained the relation between family conflict and borderline personality features in undergraduate women. To this end, family conflict (CBQ) served as the independent variable, thwarted belongingness and perceived burdensomeness (INQ) as mediators, and borderline features (PAI-BOR) as the dependent variable. This model is presented visually in Figure 1. Before testing for mediation, formal detection-tolerance and the variance inflation factor (VIF) were used to assess multicollinearity. Multicollinearity was not a problem, with tolerance greater than 0.2 and a VIF less than 4, so centering the predictor variables was not necessary [44, 45]. This multiple mediational model was tested using the Preacher and Hayes’ [46] test of the indirect effect, which allows for models in which two mediators are proposed. This method provides a bootstrap test of the indirect effect of family conflict on BPD symptomology through the proposed mediators of thwarted belongingness and perceived burdensomeness. In our model, this test confirmed the mediating effects of both thwarted belongingness and perceived burdensomeness (INQ) in the relation between conflictual family relations (CBQ) and borderline features (PAI-BOR) with the mean of the indirect effect across all bootstrap samples estimated at .58 and a resulting confidence interval that did not include 0 [*CI* = .42 and .76; 46]. Unstandardized path coefficients are presented in Figure 1.

Given the cross-sectional data, we sought to test the reverse of the aforementioned mediational models as well. Specifically, we tested two additional models: (1) family conflict mediates the relation between thwarted belongingness and borderline personality features and (2) family conflict mediates the relation between perceived burdensomeness and borderline personality features. In the first mediation, thwarted belongingness (INQ) served as the independent variable, family conflict (CBQ) as the mediator, and borderline features (PAI-BOR) as the dependent variable. Preacher and Hayes’ [46] test of the indirect effect confirmed the mediating effect of family conflict (CBQ) in the relation between thwarted belongingness (INQ) and borderline personality features (PAI-BOR) with the mean of the indirect effect across all bootstrap samples estimated at .11 and a resulting confidence interval that did not include 0 [*CI* = .06 and .16; 46]. In the second mediation, perceived burdensomeness (INQ) served as the independent variable, family conflict (CBQ) as the mediator and borderline features (PAI-BOR) as the dependent variable. Preacher and Hayes’ [46] test of the indirect effect confirmed the mediating effect of family conflict (CBQ) in the relation between perceived burdensomeness (INQ) and borderline personality features (PAI-BOR) with the mean of the indirect effect across all bootstrap samples estimated at .079 and a resulting confidence interval that did not include 0 [*CI* = .03 and .13; 46].

## Discussion

The aim of the present study was to examine the relations between family conflict, beliefs about thwarted belongingness and/or perceived burdensomeness and borderline personality features in female undergraduates. The rationale for the study was motivated by the theoretical premise that a maladaptive family environment (specifically family conflict) may be associated with beliefs about thwarted belongingness and perceived burdensomeness, which, in turn, increase vulnerability to borderline personality pathology. At the bivariate level, we found a significant relation between borderline features and conflictual family relations. This finding adds to an expanding research base that has identified the adverse family environment as an integral factor in borderline personality development [15, 27, 28, 47, 48]. Significant bivariate relations among thwarted belongingness and perceived burdensomeness and both conflictual family relations and borderline features also provide the first evidence that these beliefs may play a role in borderline personality pathology, providing highly preliminarily, cross-sectional support for the current study’s proposed theoretical model.

At the multivariate level, multiple mediational analyses revealed that the relation between family conflict and borderline personality features was explained by beliefs of thwarted belongingness and perceived burdensomeness. Given the cross-sectional nature of the data, reverse mediation was also tested to establish the directionality of these relations. Analyses revealed that family conflict also mediated the relation between thwarted belongingness and borderline personality features and that family conflict mediated the relation between perceived burdensomeness and borderline personality features. We cannot draw causal conclusions from the data due to the cross-sectional nature of the data; however, findings provide preliminary evidence that reciprocal relations may exist between family conflict, unhelpful interpersonal beliefs about thwarted belongingness and borderline features. Only through longitudinal work can the complete directionality of the potential complex and dynamic reciprocal relations be investigated.

As this represents the first study to concurrently investigate these constructs, these results cannot be directly interpreted against previous study findings; however, these results do appear to support Linehan’s [8] and others’ [6, 7, 9] developmental theoretical models of BPD. For instance, Linehan’s biosocial theory of BPD explains that neglectful, abusive, and/or dismissive family environments negate thoughts, feelings, and behaviors so that over time the child learns that his expressions and experiences are unacceptable. Ultimately, he or she adopts a pattern of self-invalidation, fostering a belief that he/she is unacceptable, defective, and unwanted. Here, the conceptual connection between self-invalidation and the development of specific beliefs about thwarted belongingness and perceived burdensomeness is easily imagined, and though this direct relation remains untested by the current study, our findings nonetheless contribute to this overarching theoretical model. Findings that reverse mediations were also significant suggest a bidirectional relation between maladaptive interpersonal beliefs and family conflict. That is, maladaptive interpersonal beliefs may both arise from and elicit family conflict. As such, a child who believes he is a burden and does not belong may evoke responses from his environment that produce family conflict, which in turn, may shape the child by reinforcing internalized maladaptive interpersonal beliefs. The design of this study precludes the examination of these causal relations; however, that these processes are likely reciprocal is consistent with Linehan’s [8] etiological model of BPD and several other models of the development of psychopathology in adolescents [49, 50].

Similarly, the application of Joiner’s [36] Interpersonal Psychological Theory to borderline personality symptomology supports theoretical and empirical conceptualizations of BPD as a highly interpersonal disorder [51–55]. This characterization reflects one of the most serious components of BPD: marked impairment in maintaining stable and healthy interpersonal relationships [1]. Gunderson and Lyons-Ruth [52] have asserted that interpersonal dysfunction arises from a core trait of “interpersonal hypersensitivity” that originates in early childhood and continues through adulthood. This is also consistent with Sharp’s hypermentalizing model of BPD in which children in a conflictual family environment become hyper-attuned to the emotions and thoughts of those around them to the extent that over interpretation of mental states occur in contexts that do not call for it [55, 56]. The extent to which beliefs about thwarted belongingness and perceived burdensomeness relate to hypermentalizing is unknown, as no studies have endeavored to examine these beliefs in this context (or in relation to BPD, in general). However, it is certainly possible that hypermentalizing would breed maladaptive interpersonal beliefs. This notion is supported by other studies demonstrating that attentional focus to social cues in BPD is related to perceptions of abandonment and defectiveness [57, 58]. Future studies should explicitly examine these interrelated constructs (i.e., hypermentalizing, interpersonal hypersensitivity and maladaptive beliefs) within the context of adverse family environments to enhance understanding of BPD’s etiology and development.

The findings of the present study are limited by several factors. Most importantly, the cross-sectional design of this study, as previously discussed, limits the interpretation of these findings by excluding causal interpretations. While this study makes a valuable first step toward understanding the interrelations between conflictual family relations, beliefs about thwarted belongingness and perceived burdensomeness, and borderline symptomology, the broad, casual theory hypothesized cannot be tested with this study design and remains a consideration for future research. Of importance here is the fact that the directionality of the relation cannot be established because all measurements were taken at one time point (thereby precluding interpretations requiring temporal precedence). Complex and reciprocal processes over time are likely to occur in which an emotionally intense child may evoke invalidating responses; and in turn, invalidating responses may evoke higher levels of intensity in the expression of emotions in already vulnerable children. Moreover, the directionality of relations identified in this study remains unknown—such that beliefs of thwarted belongingness and perceived burdensomeness could be a product of borderline symptomatology, rather than being a risk factor for BPD resulting from family conflict (as aforementioned developmental theories would suggest). A second limitation of the current study is the use of a college sample of only females, which restricts the generalizability of findings to other populations. Even more, in order to understand the disease processes underlying this disorder, and to therefore make clinical interpretations, *clinical* samples should be utilized. Another notable limitation of this study is that all measures were self-report questionnaires and therefore subject to mono-method variance, which could inflate relations among the variables examined. Finally, the use of self-report measures does not reflect evaluation of variables hypothesized to be at different levels of processing like remembered family conflict, current beliefs about thwarted belongingness and perceived burdensomeness, and behavioral and affective symptoms of BPD. Moreover, family conflict was assessed only at one time point, retrospectively, and only from the perspective of the participant. Measurement at only one time point prevents this study from determining the differential effects of current and past family conflict. Indeed, current family conflict is likely related to prior family conflict—which was not measured in the present study and therefore differential relations to borderline features and maladaptive interpersonal beliefs cannot be examined. Disentangling the effects of current and past family conflict should be a priority in future studies. Future research could improve upon these limitations by collecting multiple reports of family conflict at various time points, using an experimental task better suited to identifying maladaptive interpersonal beliefs, and assessing BPD using a diagnostic interview. Additionally, future research should evaluate other aspects of the family environment (distinct from but likely related to family conflict) like attachment security, emotional reactivity, and social cognition in relation to maladaptive interpersonal beliefs.

## Conclusions

Notwithstanding these limitations, the present study is the first to consider constructs from the Interpersonal Psychological Theory [36] as mechanisms within a developmental model of BPD. Though this theory is typically applied to depressed and suicidal cognitions, there is evidence that it may be a helpful framework for understanding cognitions at play in various psychological disorders, including BPD [37, 39]. Further, in identifying thwarted belongingness and perceived burdensomeness as mediators in the relation between family conflict and borderline symptomology, we suggest that perhaps the adverse effects of certain social-environmental factors on the development of BPD may be alleviated by interventions aimed at beliefs about thwarted belongingness and perceived burdensomeness, just as cognitive-behavioral treatments for depression and anxiety specifically target distorted cognitions central to those disorders. Therefore, the interpersonal risk factors for BPD may potentially be addressed by targeting distorted beliefs about thwarted belongingness and perceived burdensomeness in addition to family relationships, which are in some cases less amenable to change.

## Authors’ information

AK is a doctoral student at the Clinical Psychology program at the University of Houston. AV is a doctoral student at the Clinical Psychology program at the University of Houston. CS is an Associate Professor of Clinical Psychology and Director of the Developmental Psychopathology Laboratory at the University of Houston and is the Research Director of the Adolescent Treatment Program at The Menninger Clinic.
